# Engineered Gold Nanoparticles and Plant Adaptation Potential

**DOI:** 10.1186/s11671-016-1607-2

**Published:** 2016-09-15

**Authors:** Khwaja Salahuddin Siddiqi, Azamal Husen

**Affiliations:** 1 Department of Chemistry, Aligarh Muslim University, Aligarh, 202002 Uttar Pradesh India; 2 Department of Biology, College of Natural and Computational Sciences, University of Gondar, P.O. Box #196, Gondar, Ethiopia

**Keywords:** Gold, Uptake, Growth response, Productivity, Plant mechanism, Stress modulation

## Abstract

Use of metal nanoparticles in biological system has recently been recognised although little is known about their possible effects on plant growth and development. Nanoparticles accumulation, translocation, growth response and stress modulation in plant system is not well understood. Plants exposed to gold and gold nanoparticles have been demonstrated to exhibit both positive and negative effects. Their growth and yield vary from species to species. Cytoxicity of engineered gold nanoparticles depends on the concentration, particle size and shape. They exhibit increase in vegetative growth and yield of fruit/seed at lower concentration and decrease them at higher concentration. Studies have shown that the gold nanoparticles exposure has improved free radical scavenging potential and antioxidant enzymatic activities and alter micro RNAs expression that regulate different morphological, physiological and metabolic processes in plants. These modulations lead to improved plant growth and yields. Prior to the use of gold nanoparticles, it has been suggested that its cost may be calculated to see if it is economically feasible.

## Review

### Introduction

Nanomaterials are ultrafine particles whose dimensions are in the range between 1 and 100 nm. Due to their physical and chemical properties, they are being studied for their use in numerous different disciplines of science. It has the potential to revolutionise the agricultural and food industry with novel tools by enhancing the production of various plants [1–5]. Despite the fantastic potential, a number of research on the toxicological impact of engineered nanomaterials have also been performed on the plants and its surrounding environment [6–8] because thousands of tons of engineered nanomaterials are calculated to be released into air, water and soil [9]. The bioavailability and cytoxicity of nanoparticles depend on their shape, size, concentration and mobility in the aqueous medium [10–12].

Gold nanoparticles were most frequently studied and have many potential applications [5, 13]. Their interactions with animals and plants are a matter of concern [5, 7, 14]. Unrine et al. [15] have reported that gold nanoparticles are transferred from soil to invertebrates and then to the secondary consumers. Tiede et al. [6] have predicted a model for engineered gold nanoparticles in natural waters (0.14 μg L^−1^) and soils (5.99 μg kg^−1^). Thus, the study of the bioavailability and toxicity of gold nanoparticles is essential in order to assess possible risks and fate of the ecosystem. Gold nanoparticles ranged from 0.5 to 100 nm in diameter have been reported inside the many plant tissues due to metal nanoparticles exposure [16–20]. Similarly, many other nanoparticles are also translocated to the grown plantlets and get accumulated in all tissues including newly developed seeds. The germination of these seeds leads to the development of second-generation plantlets where nanoparticles are also detected in the leaves [4, 5]. They may enter the biological systems and interact with cells. In general, plant growth (root length, shoot length, biomass) enhancement has often been observed at the exposure of low concentrations of toxic metal ions and or nanoparticles [4, 5]. Quite often, hormesis is used to describe such mildly toxic stress-induced stimulatory plant growth response [21, 22]. However, inhibitory effects of higher concentrations of Au^3+^ on root growth of alfalfa and other growth attributes reduction in various plants species have also been reported [5, 23, 24]. In general, metallic gold with zero nutritive value does not cause toxicity but higher concentrations of gold solutions may cause toxicity, affect plant growth adversely [2, 18, 23, 24] and may produce changes at physiological, biochemical and molecular levels [2, 8, 25–27].

**Fig. 1:**
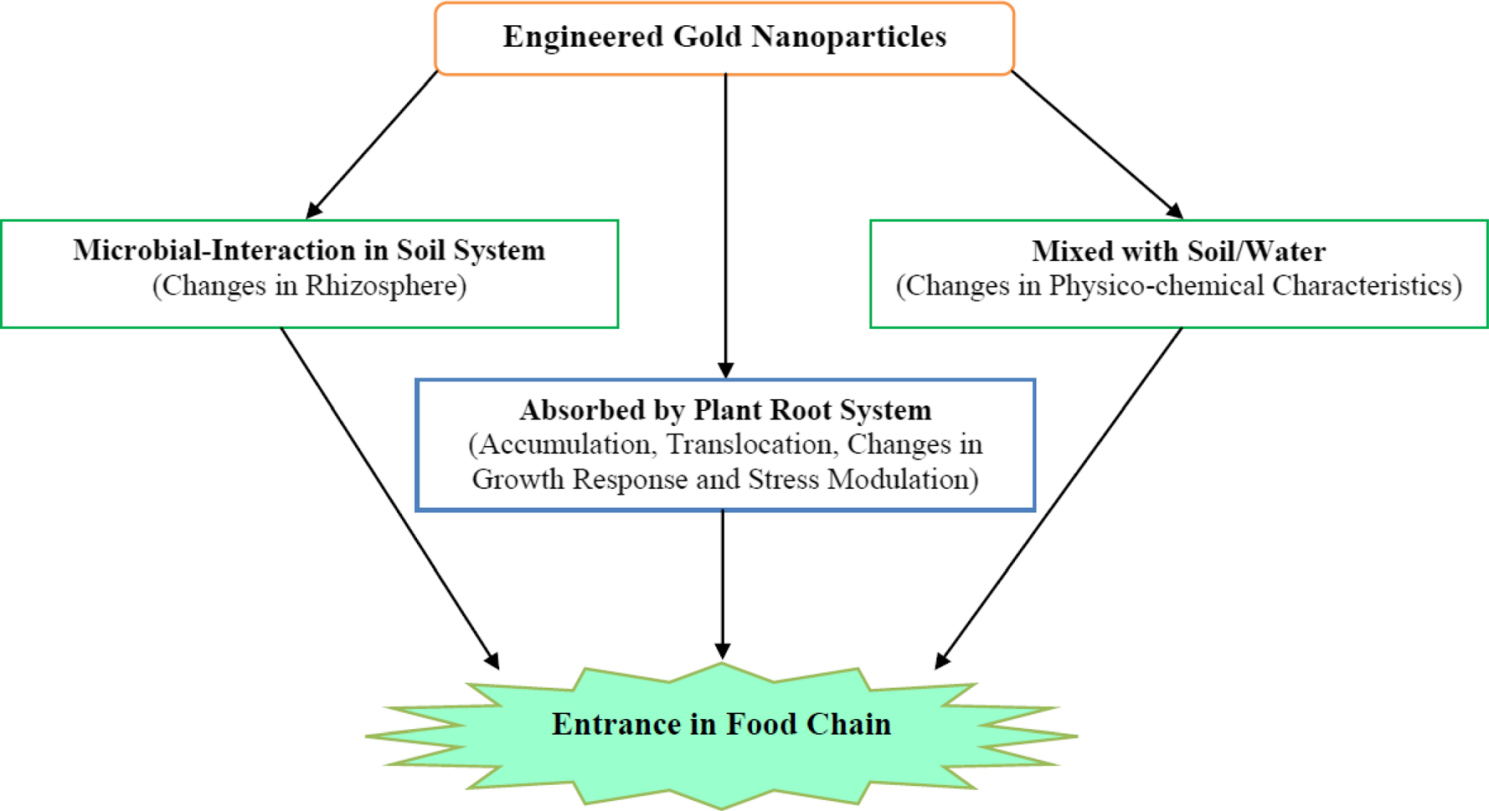
Interaction of gold nanoparticles with plant/soil system and possible entrance in food chain

The cell shape and size adjustment at the initial stages of plant growth was identified to cause severe functional impairments associated with the tissue differentiation and the solute transport [28]. Moreover, the exact mechanism of plant defence against nanotoxicity is elusive. Perhaps, the possible detoxification pathways and formation of an effective scavenging system composed of non-enzymatic antioxidants and enzymatic antioxidants might enable plants to tolerate and resist oxidative stress caused by nanoparticles [29–32]. In an experiment, Corredor and coworkers [33] have demonstrated that nanoparticles are competent to penetrate in living plant tissues, migrate to different regions of the plant system and movements over small distance are favoured. Metal nanoparticles available in aqueous medium or soil matrix may move through the symplastic or apoplastic region to penetrate the epidermis of roots, pass through the cortex, and finally translocate and distribute to the stems and leaves via the xylem and phloem [33, 34]. Thus, accordingly, the engineered gold nanoparticles can enter in the plant system and lead to favourable or undesirable changes. Further on the given facts, the fate of engineered gold nanoparticles in the ecosystem and food chain integrity are of great concern (Fig. 1).

In the present review, we focus on the role of engineered gold nanoparticles on the plant adaptation potential in terms of accumulation, translocation, growth response and stress modulation.

### Accumulation, Toxicity and Tolerance

Several studies have investigated the binding of various states of gold and gold-silver-copper alloy nanoparticles in *Medicago sativa*, *Brassica juncea* and other living plant systems [18, 35–38]. The uptake of gold nanoparticles and their effect on different plant systems have been studied and summarised in Table 1. Since plants are stationary having large size and high leaf area, they are prone to exposure to a wide range of nanoparticles available in their surrounding environment [39]. They may significantly control metals or nanoparticles by accumulating them into their biomass [40–42]. Nanoparticles enter into the soil from leaching of nano-enabled products for environmental remediation, land applications of contaminated biosolids or waste water effluent discharge [43, 44]. Availability of gold nanomaterials to plants has been investigated by Judy et al. [20]. After 7 days exposure at 30 μg gold mL^−1^ in the nutrient solution between 2 and 54 μg gold g^−1^ were detected in the dried tobacco plant. Barrena and coworkers [45] have found that there is either low or zero toxicity of gold, silver and magnetite nanoparticles at doses of 62, 100 and 116 μg mL^−1^ for cucumber and lettuce plants, respectively.

**Table 1:** Effects of engineered metal nanoparticles on plants

Nanoparticle	Size (nm)	Plant	Concentration	Effect	References
Dodecanethiol functionalized gold	–	*Lactuca sativa*	0.013 % (*w*/*w*)	No effect on the seed germination, improved shoot-to-root ratio	[68]
Gold	10	*Cucumis sativus*, *Lactuca sativa*	62, 100, 116 mg L^−1^	Positive effect on germination index	[45]
Mixture of gold/copper	–	*Lactuca sativa*	0.013 % (*w*/*w*)	No effect on the germination, improved shoot-to-root ratio	[68]
Gold	24	*Arabidopsis thaliana*	10 μg/ml, 10 and 80 μg mL^−1^	Enhance total seed yield, improved seed germination rate; vegetative growth and free radical scavenging activity	[3]
Gold	–	*Vigna unguiculata*	1 mM	No effect on growth, proline and malondialdehyde	[75]
Gold	–	*Brassica juncea*	25 ppm	Improved seed germination rate	[2]
Gold	2–19	*Hordeum vulgare*	10 μg mL^−1^	No effect on seed germination, reduced plant biomass	[24]
Gold	~25	*Gloriosa superba*	1000 μM	Improved seed germination rate, vegetative growth	[66]

Feichtmeier et al. [24] have studied the effect of spherical gold nanoparticles of 2–19 nm on barley seed germination. There was no significant effect on germination, but yellowing of leaves, darkening of roots and decreased biomass were observed which further deteriorated with increasing concentration of gold nanoparticles. However, the very low concentration of 1 μg gold mL^−1^ of gold nanoparticles in the nutrient medium had a growth-stimulating effect. It is thought that perhaps low dose activates the functioning of hormones [45] whilst larger doses and larger nanoparticles negatively influenced on plant growth and biomass production, perhaps adsorption of gold nanoparticles onto the cell wall surfaces of the primary root system, diminished the pore size and thus inhibiting water transport capacity, which ultimately decreased plant growth and related attributes. This was also explained by previously workers [24, 46]. Reduced plant growth under water stress conditions has also been reported by various workers [21, 47–49]. Formation of monodispersed spherical gold nanoparticles in roots of *Arabidopsis* grown hydroponically in the presence of 10 ppm of KAuCl_4_ was observed [26]. Its yield was also found to be plant organ dependent, for example, shoot extract of *Cucurbita pepo* produced large number of spherical nanoparticles of smaller size than those produced from roots [27]. In order to understand the formation of metal nanoparticles in plants, *Brassica juncea* was hydroponically grown in the presence of AgNO_3_ and HAuCl_4_ separately [50]. The plant was cut into different sections and analysed by various techniques. From X-ray absorption spectroscopy, silver and gold nanoparticles were found to be deposited in leaves, stem, roots and cell wall of *B. juncea*. Spherical silver and gold nanoparticles of 2–100 nm were formed as a consequence of reduction of metal by reducing sugars shown below:

$$ \begin{array}{lll}\mathrm{A}\mathrm{g}\mathrm{N}{\mathrm{O}}_3\hfill & \to \hfill & \mathrm{A}{\mathrm{g}}^{+} + \mathrm{N}{{\mathrm{O}}_3}^{-}\hfill \\ {}\mathrm{A}{\mathrm{g}}^{+} + \mathrm{reducing}\ \mathrm{sugar}\hfill & \to \hfill & \mathrm{A}{\mathrm{g}}^0\hfill \\ {}\mathrm{H}\mathrm{A}\mathrm{u}\mathrm{C}{\mathrm{l}}_4\hfill & \to \hfill & \mathrm{H}\mathrm{A}\mathrm{u}\mathrm{C}{\mathrm{l}}_3 + \mathrm{H}\mathrm{C}\mathrm{l}\hfill \\ {}\mathrm{A}\mathrm{u}\mathrm{C}{\mathrm{l}}_3\hfill & \to \hfill & \mathrm{A}{\mathrm{u}}^{3+} + 3\mathrm{C}{\mathrm{l}}^{-}\hfill \\ {}\mathrm{A}{\mathrm{u}}^{3+} + \mathrm{sugar}\hfill & \to \hfill & \mathrm{A}\mathrm{u}\hfill \end{array} $$

**Fig. 2:**
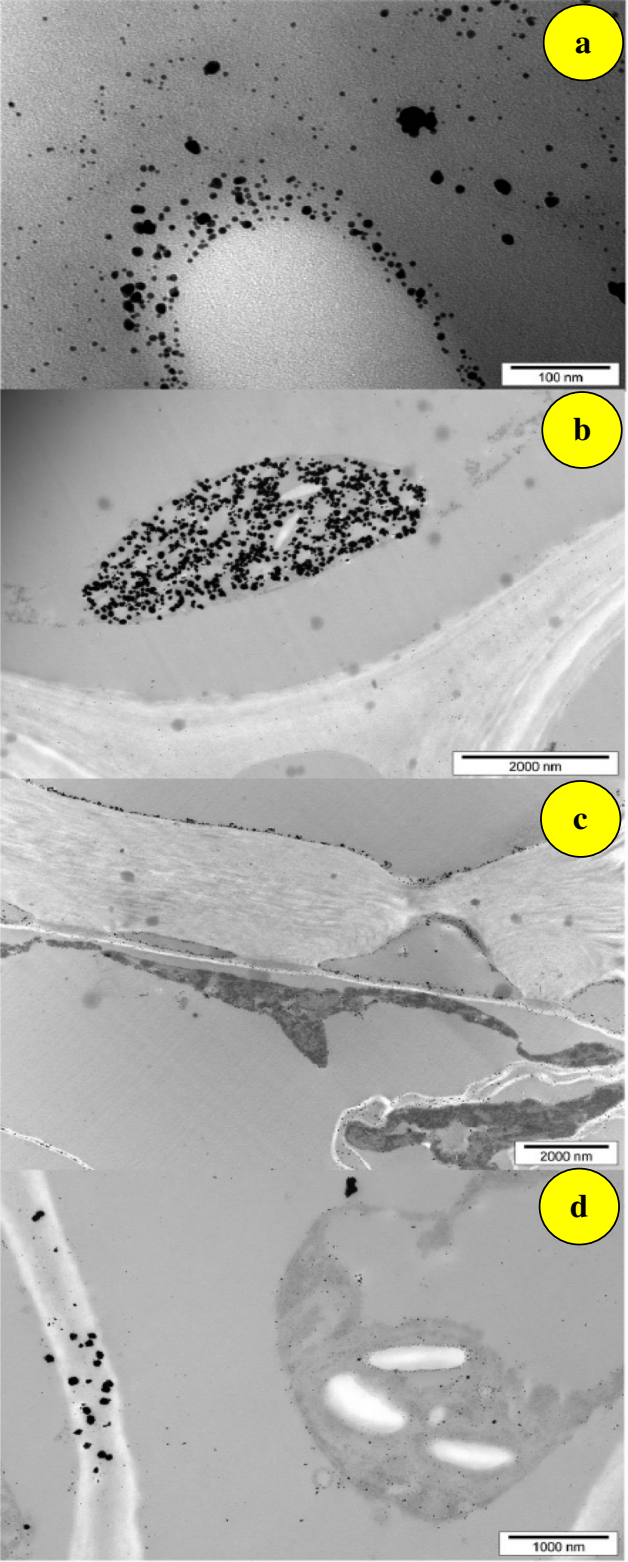
Gold nanoparticles in *Brassica juncea*. **a** Leaf. **b** Stem. **c** Root. **d** Leaf cell wall [50]

These nanoparticles are used in catalysis, drug delivery and photonics [51]. The metal deposition and sequesterization may be used in phytomining and phytoremediation [42, 52]. This is also a way to remove toxic metals from sewage, sludge and garbage. Redox reaction has been recognised in the regulation and prevention of toxic metals from spreading. The formation of nanoparticles from the metal salts and minerals absorbed by the plants is dependent on the reducing potential of certain plants. For instance, the presence of glucose, fructose, proteins, phenols and amino acids in different parts of the plant acts as reducing agents. The minerals present in the plants are therefore reduced to metal nanoparticles if sufficient amounts of reducing substances, mentioned above, are present. The transmission electron microscopy (TEM) image of the plant material treated with AgNO_3_ and HAuCl_4_ shows dense deposit of gold and silver nanoparticles. The plant tissue also showed damage and degradation of the cell wall (Fig. 2). The metal nanoparticles thus formed were found to be deposited in all parts of the plant, although they were in abundance around the chloroplasts. Nanoparticles are sometimes associated with starch granules in the chloroplast. Perhaps starch also acts as a reductant that is why nanoparticles are found around them. Beattiew and Haverkamp [50] have shown that the *B. juncea* grown in HAuCl_4_ contains gold nanoparticles of 2–100 nm in different parts of the plants. They are mainly spherical but triangles, diamonds and hexagons have also been detected. The larger nanoparticles form agglomerates in leaves and occasionally in stems.

It is clear that high concentration of nanoparticles are accumulated in and around the chloroplast because they are the production centre and storehouse of sugars produced during photosynthesis. Incidentally, *B. juncea* contains reducing sugars such as glucose and fructose [53]; therefore, it is believed that they reduce AgNO_3_ and HAuCl_4_ salts to silver and gold nanoparticles, respectively. Reducing sugars are tested by Benedicts and Fehling’s reagents which involve the reduction of Cu^2+^ to Cu^+^ which has a standard reaction potential of +0.16 V [54]. It is obvious that any metal salt may be reduced to metal provided the reduction potential of the metal ion to metal is greater than +0.16 V.

$$ \begin{array}{ccc}\hfill {\mathrm{M}}^{\mathrm{n}+}\hfill & \hfill \to \hfill & \hfill \mathrm{M}\hfill \end{array} $$

All precious metal salts to metal reduction potential ranges between 0.35 and 1.0 V. The metals include Au, Ag, Ir, Pt, Pd, Rh, Ru and Cu which may be reduced by reducing sugars present in *B. juncea* because the reduction potential is within the above range. The reduction of metal salts continue to yield metal nanoparticles till all the reducing sugars are completely consumed.

It has been reported that *Chilopsis linearis* plant tissues are able to take up gold from gold-enriched media (160 mg Au L^−1^ in agar) and synthesised nanoparticles of average size of 8, 35 and 18 Å in root, stem and leaves, respectively. The average size of the gold nanoparticles in various tissues has been observed to be related to the concentration of gold in different parts in the plant [17]. This intracellular nanoparticle synthesis ability revealed its possible use for phytoextraction applications. Nonetheless, with the same plant, very small size nanoparticles (0.55 nm) might also be synthesised [36]. Also, growth of *Sesbania* seedlings in chloroaurate solution resulted in the accumulation of gold with the formation of stable gold nanoparticles in plant tissues. TEM study exhibited the intracellular distribution of monodispersed nanospheres. It seems that the reduction of the metal ions was catalysed by secondary metabolites present in the cells [18].

Practically, gold is not an element required by plants as a trace element; however, the accidental absorption or insertion may often show some dramatic change in plant growth. Taylor and coworkers [8] have shown the effect of gold/gold nanoparticles on the physiological and genetic responses of *Arabidopsis thaliana* that its root length was reduced by 75 % at 100 mg L^−1^ concentration. Gold ions were detected in roots and shoots, but gold nanoparticles were absorbed only in the root tissues of the plant. Upregulation of genes involved in the plant stress response like glutathione transferases, cytochromes P450, glucosyl transferases and peroxidises have been observed. Similarly, downregulation of gene-encoding proteins involved in the transport of copper, cadmium, iron and nickel ions and aquaporins bonded to gold have been found. Gold disrupts protein structure and may also displace some essential metal nutrients from proteins.

Gold nanoparticles of 0.5 to 100 nm diameter have been found in the tissues of plants exposed to metal nanoparticles [17, 36, 55]. It is suggested that nanoparticles are first oxidised to Au^+^ or Au^3+^ and transported to the tissues followed by their reduction to gold nanoparticles.

$$ \begin{array}{lllll}\mathrm{Gold}\ \mathrm{nanoparticles}\hfill & \overrightarrow{\mathrm{Oxidation}}\hfill & \mathrm{Gold}\ \mathrm{ions}\hfill & \overrightarrow{\mathrm{Reduction}}\hfill & \mathrm{Gold}\ \mathrm{nanoparticles}\hfill \end{array} $$

The question arises why take such a long process to oxidise and then reduce to gold nanoparticles. In such cases, the plants must contain both oxidising and reducing compounds and it would take a long time to complete the cycle. However, it appears most probable that once the gold ions are reduced to nanoparticles, they are transported to the shoots and leaves.

Gold nanoparticles coated with polymers are stabilised and released slowly thereby reducing their toxicity and agglomeration. Furthermore, the uptake and incorporation of nanoparticles into the plant cells have been discussed by many researchers [5, 24, 56–58]. Penetration of gold nanoparticles into the protoplasts by endocytosis was found to be linked to different pathways [59]. Their penetration through lipid membranes bypassing endocytosis has also been reported. TEM images exhibited the presence of gold nanoparticle in the cytoplasm of root cells of *Sesbania drummondii* [18]. Rodriguez et al. [17] have hypothesised that gold nanoparticles in the solution are associated with the carboxylic acid moieties present in the cell wall and thus once the gold particles enter root cells, they are transported symplastically to the aerial parts of the plant. *S. drummondii* exhibits intracellular formation of nanomaterials perhaps because of metal ion reduction by secondary metabolites present in the cells. Recently, it has been demonstrated that tomato can uptake gold nanoparticles without altering its properties [60]. In an experiment, Zhai and coworkers [57] used poplar plants (*Populus deltoides* × *nigra*, DN-34) to explore the vegetative uptake of gold nanoparticles and their subsequent translocation and transport into plant cells. They treated total gold concentrations in the leaves of plants with 15-, 25- and 50-nm gold nanoparticles at exposure concentrations of 498 ± 50.5, 247 ± 94.5 and 263 ± 157 ng mL^−1^ in solutions with 0.023 ± 0.006, 0.0218 ± 0.004 and 0.005 ± 0.0003 μg g^−1^ of dry weight, respectively, which accounted for 0.05, 0.10 and 0.03 %, respectively, of the total gold mass added. Redox process of converting Au^3+^→Au^0^ was confirmed from a change in colour from yellow to pink in the hydroponic solution containing the stem of poplar plant. Nearly 90 % Au^3+^ ions were reduced to Au^0^ in 2 days. It is now believed that all metal nutrients in ionic form are slowly reduced to metallic form and transported to various parts of the plant. They have demonstrated that the Au^3+^ ions were absorbed by the roots of poplar and reduced them to gold nanoparticles without dissolving in Au^3+^ ions. Thereafter, the gold nanoparticles were observed in the cytoplasm and various organelles of the root and leaf cells. The aggregation of gold nanoparticles in the plasmodesmata might affect the transport of nutrients and other materials from companion cells, which may produce the toxic effect observed on poplar plants. In addition, authors also found the presence of gold nanoparticles inside the leaves and mainly existed in the xylem suggesting that gold nanoparticles also followed the transmission route of water and nutrients through the xylem to the leaves.

**Fig. 3:**
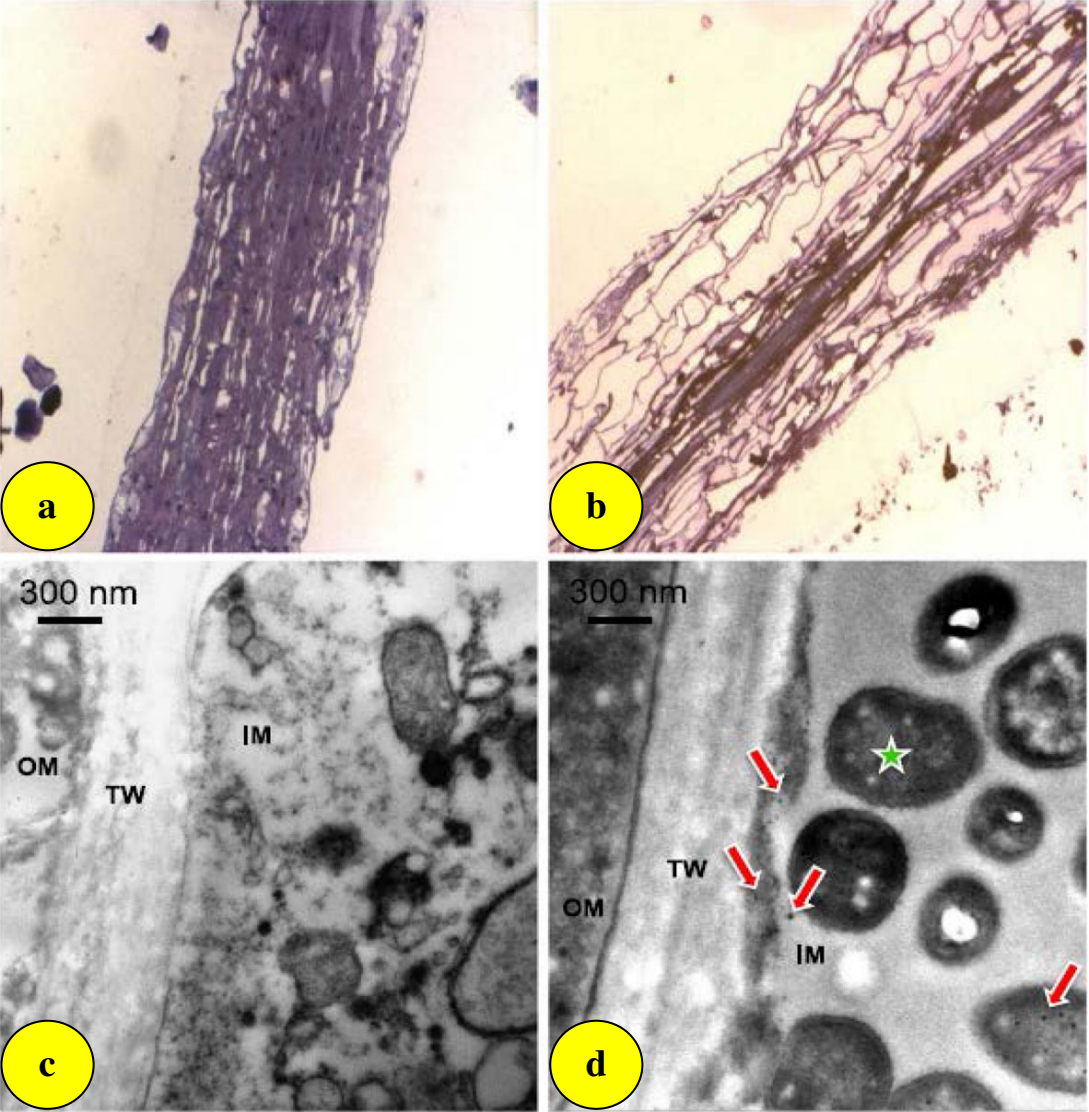
Images of barley (*Hordeum vulgare*) primary root tips. Light microscopic observation (magnification, ten times) of longitudinal sections of barley primary root tips of (**a**) control plants and (**b**) plants exposed to 10 μg mL^−1^ gold nanoparticles; TEM images of root cross-sections of (**c**) control plants and (**d**) plants exposed to 10 μg mL^−1^ gold nanoparticles. Bacteria (*asterisk*) and gold nanoparticles (*arrows*). *OM* outer matrix, *IM* inner matrix, *TW* thick wall [24]

In a recent study, Feichtmeier et al. [18] have found clear morphological changes in the root tips of *Hordeum vulgare* exposed to gold nanoparticles. The epidermis was broken and the cortical cells collapsed. TEM images of exposed samples have also confirmed the penetration of gold nanoparticles of 18 nm diameter suggesting their accumulation in the root (Fig. 3). Similarly, 4-nm gold nanoparticles accumulated in root cells of freshwater plants [61]. Gold nanoparticles penetration in tobacco plants through the root cells has also been examined [62]. It has been suggested that gold nanoparticles enter to tobacco plants through size-dependent mechanisms, translocate to plant cell system and produce toxic symptoms [62]. In poplar plants, the size distribution of the gold nanoparticles appears to be somewhat modified during uptake and transport with the exception of the 50-nm gold nanoparticles, which maintained their diameter and were surrounded by some unknown substances in the leaf xylem cells [57]. In this study, gold nanoparticles were found more in the roots than in the leaves because the roots directly contacted gold nanoparticles in the treatment solution, whilst only a small fraction of the gold nanoparticles was observed in the leaves. Thus, accumulation and translocation of gold nanoparticles in different parts of a plant are not uniform. Gardea-Torresdey et al. [36] proposed that the roots absorb the dissolved minerals by capillary action and subsequently reduced them to metal nanoparticles. In addition, the uptake of nanoparticles appears to be size dependent as aggregates of gold nanoparticles of 3.5 nm were seen in the root cytoplasm of tobacco but larger (18 nm) ones were not absorbed [62]. The absorption of nanoparticles also varies from species to species [41, 63]. Zhu et al. [64] have suggested that gold nanoparticles’ uptake and distribution depends on both nanoparticle surface charge and plant species.

### Plant Growth Response and Modulation for Detoxification

**Fig. 4:**
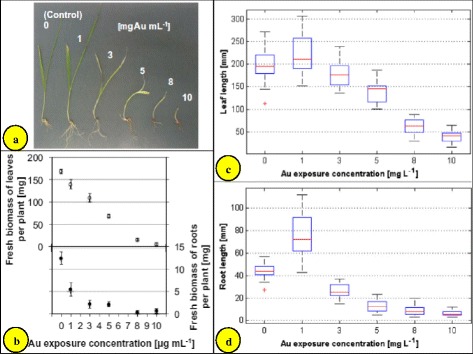
Concentration-dependent effects of gold nanoparticle exposure on *Hordeum vulgare* after 2 weeks of exposure. **a** Photos of barley seedlings, **b** fresh biomass of leaves (*empty symbols*) and roots (*filled symbols*) of barley plants (mean ± one standard deviation for three individual batches with four to ten barley seedlings each), **c** leaf lengths, and **d** root lengths of barley plants (*box plots* represent median values including 14–21 individual data sets per concentration; *bars* give minimum and maximum values excluding outliers marked as *red crosses*) [24]

Random use of metal nanoparticles in plants or food crops may not produce desired vegetative growth or enhance the yield of food crops. It must be known that which trace elements are useful for the plant so that the same nanoparticles may be used. Several investigators have demonstrated that gold can be accumulated, to varying degrees, by plant species including *B. juncea*, *B. campestris*, *Trifolium repens*, *Sorghum helense*, *Raphanus sativus*, *Kalanchoe serrate* and *Helianthus annuus* [65]. Studies related to gold nanoparticle exposure and plant growth have been reported in the recent years [20]. For example, gold nanoparticle exposure improves seed germination in lettuce, cucumber [46], *B. juncea* [2] and *Gloriosa superba* [66]. The foliar spray of gold nanoparticles on *B. juncea* seedlings showed changes both in growth (height, stem diameter, number of leaves, number of branches, number of pods) and yield of seed [2]. The oil, reducing sugar and total sugar contents also increased. Authors also claimed that the foliar spray of gold nanoparticles improved the redox status of the treated plants. The per cent germination increased when *B. juncea* seedlings were sprayed/inoculated with 25 ppm gold nanoparticles. However, the germination rate was decreased at the higher concentration of gold nanoparticles. The authors have suggested that the antagonistic effect of gold nanoparticles slows down the effect of ethylene; as a result of which, an increase in the number of leaves of *B. juncea* occurs. In fact, it is not the antagonism of gold nanoparticles but the complexation of ethylene with gold or adsorption of ethylene on gold nanoparticles. An average 19 % increase in the seed of *B. juncea* was noted after treating the plant with about 10 ppm gold nanoparticles. However, it is not economically feasible as the cost of gold nanoparticles (10 mg L^−1^) sprayed seems greater than the yield of the crop; nevertheless, it is an attempt towards a bright future for increased food crop produced with engineered gold nanoparticles. Fresh biomass of *H. vulgare* decreased, the leaves turned yellow and the root turned dark brown (Fig. 4) with increasing concentration (0–10 mg L^−1^) of gold nanoparticles [24].

Several researchers have reported that the gold nanoparticles inhibit plant growth [8, 17, 18, 23, 67, 68] but the exact mechanism of inhibition is not well understood. In order to withstand the harsh impacts of the reactive oxygen species (ROS), plant systems are known to evolved antioxidant defence mechanisms that involve enzymatic as well as non-enzymatic components and operate to alleviate oxidative damage and enhance plant resistance to stress conditions [47, 48, 69] or against any kind of foreign matter [63]. Exposure of gold nanoparticles from 100 to 400 ppm decreases plant growth and was attributed to increase in free radical stress, supported by a general increase in antioxidative enzymes such as ascorbate peroxidase, catalase, guaiacol peroxidase and glutathione reductase. In addition, proline (osmolyte) and hydrogen peroxide were also increased due to the formation of ROS. These findings have indicated that production of ROS depends on the concentration of gold nanoparticles, which impose physiological and biochemical stress over the seedlings of *B. juncea* [70]. Therefore, at higher concentration of gold nanoparticles, ascorbate peroxidase, catalase, guaiacol peroxidise and glutathione reductase were increased. Aquaporins (a group of integral membrane proteins) are water channels that not only selectively allow water molecules to flow in and out of the tissue but also reject certain substances in order to maintain the equilibrium. Shah and Belozerova [68] have shown that gold nanoparticles induce toxicity in plants by inhibiting aquaporin function. In general, loss of plant productivity due to stress is an outcome of imbalance in cellular ionic and osmotic balances which restrict photosynthesis by decreasing the abundance of green pigments causing stomatal closure and oxidative stress, thereby resulting in the formation of ROS such as superoxide, hydrogen peroxide, hydroxyl radical and singlet oxygen, which can damage the mitochondria and chloroplasts by disrupting cellular structures [47, 69, 71, 72]. ROS deteriorate membrane function, enhance membrane lipid peroxidation and cause electrolyte leakage [47, 72]. Marambio-Jones and Hoek [73] have reported that biomolecules such as proteins, glycoprotein, lipids, fatty acids, phenols, flavonoids and sugars strongly control the free radical formation. Arora et al. [2] have shown enhancement in the growth of *B. juncea* seedlings treated with gold nanoparticle (10 ppm). However, with further increase in the concentration of gold nanoparticles from 10 to 100 ppm, the level of malondialdehyde was increased which may reduce the oxidative load at higher concentrations. In this study, a 29 % higher hydrogen peroxide was recorded with the 100-ppm gold nanoparticle solution. Since hydrogen peroxide is associated with defence-related responses in plants, the increase in hydrogen peroxide level at higher concentration of gold nanoparticle solution could help to induce detoxification and protective mechanism in *B. juncea* seedlings. In *A. thaliana*, gold nanoparticles showed a significant role in seed germination, antioxidant system and altered levels of microRNA expression that regulates different morphological, physiological and metabolic processes [3].

Jain et al. [26] have reported the dose-dependent effect of KAuCl_4_ on primary root length of *Arabidopsis* seedling. It was observed that the treatment of the root with 10 ppm KAuCl_4_ triggered a significant increase in length, but at higher concentrations (25, 50 and 100 ppm), a significant decrease occurred. Obviously, low dose of KAuCl_4_ stimulates the root growth whereas higher doses inhibit it. It has been referred to the production of ROS or stress-induced antioxidants [74]. Also, at higher doses, iron is depleted and an increase in zinc and phosphate contents occurs. As a consequence of decrease in iron content in root tips and increase in monodispersed gold nanoparticle, an increase in iron-responsive genes is triggered [26].

Response of gold nanoparticles exposure on growth, yield and expression profile of microRNA of *A. thaliana* has been studied [3]. *A. thaliana* exposed to 80 and 10 μg mL^−1^ of gold nanoparticle showed flowering 15 and 5 days earlier than the non-treated plants. The number of seed per pod, the pod length and total number of seeds were much higher in plants treated with 10 μg mL^−1^ of gold nanoparticle than those treated with 80 μg mL^−1^. Increasing concentration of nanoparticles above 10 μg mL^−1^ does not increase the growth or yield of the plants. Gold nanoparticle exposure of seedlings with 10 and 80 μg mL^−1^ showed 3.54- and 2.59-folds higher inhibition of ROS which suggests the free radical scavenging efficiency of gold nanoparticle. The activity of antioxidant enzymes, namely ascorbate peroxidase, catalase, glutathione reductase and superoxide dismutase, was significantly increased.

In cowpea, phenols have been reported to impart Au(III) tolerance by the generation of gold nanoparticles in the culture medium [75]. Authors have studied the impact of gold nanoparticles on the germination and shoot and root length of cowpeas. It is surprising that 1-mM solution of HAuCl_4_ did not alter any physiological property of cowpeas. Also, it had no visible influence on growth and weight of the root/shoot. The toxic metals such as Cd^2+^, Zn^2+^, Co^2+^ and Pb^2+^ inhibit plant growth [42, 76]. They exhibited enhanced level of stress markers like protein MDA (a cytotoxic by product of lipid peroxidation) which suppress the growth. It is due to lipid peroxidation by ROS which is produced as a consequence of suppression of the electron transport system. It is therefore suggested that plant and seedlings have potential to tolerate 1 mM concentration of HAuCl_4_ or it is within the permissible limit which does not produce any adverse effect in cowpea seedlings. However, when the seedlings were raised in the HAuCl_4_ solution, the yellow colour turned purple as a result of the formation of gold nanoparticles which showed an intense absorption at 550 nm due to its surface plasmon resonance [77, 78]. Intensity of the peak in the UV-vis region increases with increasing concentration of HAuCl_4_ yielding gold nanoparticles. TEM images of its colloidal solution showed crystalline nanoparticles of the 20–50-nm gold nanoparticles.

In a study, Bekkara et al. [79] observed that the aqueous solution of the germinating cowpea seedlings turned brown due to the release of phenols, but in the presence of HAuCl_4_, it turned purple due to the production of gold nanoparticles. It is believed that it is a defensive mechanism of the plant which turns Au^3+^ ions to gold nanoparticles in the presence of phenols. The plant releases phenols even in the absence of HAuCl which may be oxidised to polyphenols. However, the reduction is essentially due to phenols which are converted to quinones leading to the formation of gold nanoparticles. It was observed that cowpea seedling with seed coat produced five to sixfold higher quantity of gold nanoparticles than those exposed to HAuCl_4_ without seed coat. It suggests that seed coat contains larger amount of phenols which helps in the rapid reduction of Au^3+^ to gold nanoparticles. Interestingly, suppression of plant growth occurs only when HAuCl_4_ concentration exceeds 0.1 mM.

## Conclusions

Nanotechnology is an emerging discipline of sciences, and gold nanoparticles have many potential applications. Use of engineered gold nanoparticles in plant production and improvement has shown remarkable promising potential. Both positive and negative impacts of gold nanoparticles in plant system have been observed. They enter into plants through a size-dependent mechanism where they may trigger the growth/biomass or inhibit the growth by causing an imbalance at physiological, biochemical and molecular levels producing oxidative stress. Gold nanoparticle exposure showed higher inhibition of ROS which suggests the free radical scavenging efficiency of gold nanoparticle. The activity of antioxidant enzymes was also increased and facilitated stress modulation to plants. Their exposure to plants has also altered microRNA and gene expression. Gold nanoparticles may be applied in fruiting plants to increase the quality and quantity of the fruits and vegetable. Prior to its use, the cost may be calculated to see if it is economically feasible. Furthermore, increasing production of engineered gold nanoparticles, use and disposal will inevitably lead to their release into the ecosystem. Thus, there is a need of systematic investigation to evaluate the impact of gold nanoparticles on plant system and their surrounding ecosystem.
